# Fast-track hip and knee arthroplasty – have we reached the goal?

**DOI:** 10.1080/17453674.2018.1550708

**Published:** 2018-12-05

**Authors:** Thomas W Wainwright, Henrik Kehlet

**Affiliations:** a Orthopaedic Research Institute, Bournemouth University, UK;; b Section of Surgical Pathophysiology, Rigshospitalet, Copenhagen University, Denmark and The Lundbeck;; c Foundation Centre for Fast-track Hip and Knee replacement, Copenhagen, Denmark.

Total hip arthroplasty (THA) and total knee arthroplasty (TKA) are common major surgical procedures that are often performed in older patients with complex comorbidities. Fast-track programs (or Enhanced Recovery after Surgery (ERAS) programs) have evolved during the past 20 years, and have been proven to reduce length of hospital stay (LOS), morbidity, and convalescence time, without an increase in readmission rates or compromising patient safety (Kehlet 2013, Khan et al. 2014, Berg et al. 2018, Petersen et al. 2018a). So far so good. However, even though patients may meet discharge criteria within 0–2 days, they have not completely recovered. In addition, despite the scientific evidence for enhanced recovery, widespread implementation is still lagging, since LOS is still around 4–6 days in many places after THA and TKA compared with 2–3 days or less in large epidemiological studies (Khan et al. 2014, Berg et al. 2018, Petersen et al. 2018a). Therefore, the challenge is to further understand the pathophysiological mechanisms of morbidity and recovery, and to optimize post-discharge functional outcomes, in order to prevent sub-acute problems turning into chronic problems.

Pain during early postoperative recovery is multifactorial with several contributors been identified, e.g. postoperative inflammatory/immunological responses, sufficient pain management and organizational factors (Kehlet 2013, Gaudilliere et al. 2014). Optimal pain management is a prerequisite to enhance recovery and plenty of analgesic techniques have been described for THA and TKA, but without firm conclusions and recommendations (Karlsen et al. 2015, 2017, Soffin et al. 2018a, 2018b). Simple multimodal oral analgesia supplemented by a high dose of preoperative glucocorticoid is probably the simplest and safest option to enhance analgesia and early recovery (Kehlet and Lindberg-Larsen 2018), with use of local infiltration analgesia (LIA) in TKA but without the use of more specific peripheral nerve blocks. The latter seemingly provide better immediate postoperative pain relief, but have not led to shorter LOS, nor have they demonstrated long-lasting improved recovery.

Since early ambulation is essential, the problem of early postoperative orthostatic intolerance is important, and studies have shown an undesirable shift in autonomic nervous system function towards increased parasympathetic function and loss of sympathetic stimulation, especially to the lower legs (Jans and Kehlet 2017). So far, the problem has not been solved by optimized fluid management, while some positive data using an α-1 agonist (midodrine) needs further study (Jans and Kehlet 2017).

Recently, more emphasis has been put on the role of pre- and postoperative anemia as risk factors for a prolonged hospital stay and re-admission after THA and TKA, clearly documenting the need for the effective preoperative diagnosis and treatment of anemia (Munoz et al. 2017). Intraoperative blood loss should be decreased by use of combined local and systemic tranexamic acid treatment, while the influence of postoperative anemia remains to be explored further especially in elderly patients and with a focus on cardiovascular complications and functional recovery. In addition, studies are required to evaluate the optimal transfusion triggers in specific high-risk patients, which unfortunately have not been included in previous THA and TKA transfusion guidelines due to lack of sufficient data (Munoz et al. 2017, 2018, Petersen et al. 2018a).

Postoperative delirium and cognitive dysfunction are well-recognized problems after surgery in general where the pathogenesis is again multifactorial and includes pain, use of opioids, sleep disturbances and inflammatory responses (Kehlet 2013). Recent large-scale data from fast-track THA and TKA have shown virtually no risk of early delirium (Petersen et al. 2017). Nevertheless, postoperative sleep disturbances (Kehlet 2013) are still present and may also be related to post-discharge functional recovery where the combination of pain, function and sleep disturbances are dominant and where future studies should clarify the relative role of each component (Krenk et al. 2010).

The final challenge lies in safety as a further reduction of morbidity and mortality, which is well documented in fast-track THA and TKA (Kehlet 2013, Khan et al. 2014, Jorgensen et al. 2016). An effort to construct a prediction risk including all conventional risk factors showed that this could be done with statistical significance, although the clinical significance was less due to the low numbers of complications, especially when separated between direct “medical” and “surgical” complications (Jorgensen et al. 2016). Part of the problem is that not only preoperative risk factors are important, but also the perioperative care regimes including avoidance of postoperative anemia. Although there is general agreement that fast-track THA and TKA decreases the risk of “medical” complications, the concern that early discharge may lead to more “surgical” complications (hip dislocation, wound problems, etc.) has not been supported by the literature (den Hartog et al. 2013, Khan et al. 2014, Jorgensen et al. 2016, Sutton et al. 2016, Pamilo et al. 2018).

Recent evidence points to patients with preoperative “psychiatric” conditions on psychopharmacological treatment – mostly with conventional antidepressants – as being at risk for prolonged LOS and more readmissions. Detailed analysis suggests that these recovery issues are related to the psychopharmacological treatment per se rather than the “psychiatric” condition, calling for future interventional studies in such patients (Gylvin et al. 2017).

The risk of post THA/TKA thromboembolic complications is well known and has been a classical model in thromboembolic prophylactic studies. However, even the most recent guidelines (NICE 2018) are predominantly based on studies with prolonged LOS and thus not reflective of the benefits of early postoperative mobilization (Kjaersgaard-Andersen and Kehlet 2012). More recent prospective data from large cohort studies suggest that conventional prolonged thromboembolic prophylaxis may not be necessary within a successful enhanced recovery THA/TKA program including early mobilization (Kehlet 2013, Petersen et al. 2018b); this needs corroboration with further large-sized studies.

Whilst early ambulation and reduced LOS has reduced the risk of complications, the early loss of muscle function may delay post-discharge recovery. Despite considerable efforts to improve rehabilitation, the use of preoperative exercise, conventional physiotherapy regimes, and earlier initiated and more intense postoperative strengthening regimes have all been found to have a limited effect in the “average” patient (Bandholm et al. 2018). Therefore, future work in fast-track THA and TKA must reveal which therapeutic interventions are effective, and in which patients with known preoperative indicators of likely delayed recovery such as pain status, frailty, psychological status, socioeconomic status, and unrealistic expectations of recovery. Although patient-reported outcome measures (PROMs) show improvement in the majority of fast-track THA and TKA patients, discrepancies are seen when compared with objective measures of functional performance and physical activity, both in the early and later recovery phase (Luna et al. 2017, 2018). Consequently, objective functional data are important from future studies, given the known increased healthcare costs and lower income levels of patients even after fast-track THA and TKA (Kjellberg and Kehlet 2016).

Although LOS in several countries has been reported to be short (Kehlet 2013), the aim is to improve recovery to be able to go directly home and not via another institution such as nursing care facilities, rehabilitation homes etc. (Cram et al. 2018) where limited scientific information is available with regard to recovery interventions and results. The same applies to the more recent publications on outpatient THA and TKA, often from private and semi-private institutions. So far, this approach seems safe in selected patients without increasing risk of readmissions or complications (Vehmeijer et al. 2018). However, despite this progress, there are challenges to understand how to increase the percentage of patients recovered and discharged on the day of surgery, and for which patients that outpatient surgery may not equate to optimized care. Fast-track protocols have been based on the concept of “first better – then faster,” so it could be that for some identified patients a planned longer stay in hospital is the best means of accelerating recovery and reducing complications, re-admissions, and morbidity.

In summary, the fast-track THA and TKA approach has made major progress, but we have not achieved the final goal. Future challenges lie in: (1) preoperative prediction of high-inflammatory responders, (2) further dose-finding or repeat-dosing glucocorticoid or other anti-inflammatory agents in studies in hyper-inflammatory responders (Kehlet and Lindberg-Larsen 2018), (3) more focused studies on high-pain responders (preoperative opioid users, pain catastrophizers, sensitized patients, etc.) (Gilron et al. 2018), and (4) developing optimal post-discharge rehabilitation strategies. Only then we will have succeeded ino reaching the goal for the “pain and risk free” THA and TKA (Kehlet and Jorgensen 2016).

## Competing interests

None.

**Thomas W Wainwright** *Orthopaedic Research Institute, Bournemouth University, UK* **Henrik Kehlet** *Section of Surgical Pathophysiology, Rigshospitalet,* *Copenhagen University, Denmark and The Lundbeck* *Foundation Centre for Fast-track Hip and Knee replacement, Copenhagen, Denmark.* *email:henrik.kehlet@regionh.dk*
